# The Impact of COVID‐19 on Share Prices in the UK*

**DOI:** 10.1111/1475-5890.12226

**Published:** 2020-07-06

**Authors:** Rachel Griffith, Peter Levell, Rebekah Stroud

**Affiliations:** ^1^ University of Manchester; Institute for Fiscal Studies; ^2^ Institute for Fiscal Studies; ^3^ Institute for Fiscal Studies

**Keywords:** share prices, coronavirus, COVID‐19

## Abstract

The spread of COVID‐19, and international measures to contain it, are having a major impact on economic activity in the UK. In this paper, we describe how this impact has varied across industries, using data on share prices of firms listed on the London Stock Exchange, and how well targeted government support for workers and companies is in light of this.

In this paper, we describe how the impact of COVID‐19 has varied across industries, using data on share prices of firms listed on the London Stock Exchange, and how well targeted government support for workers and companies is in light of this. This follows Ramelli and Wagner (2020), who describe the impact on the US and China by looking at changes in share prices, and Gormsen and Koijen (2020), who provide further analysis of the US.

Share prices convey important information on market expectations on firms’ current and future profitability. Buying a share (or stock) makes the purchaser a part‐owner of a company. Shareholders get a vote in certain corporate decisions (for example, to select members of the company board) and, perhaps more importantly, they are also paid a share of the company's profits (dividends).

Importantly, the value that shares are trading for in the stock market tells us not only about how well a company is doing today but also about how well it is expected to do in the future. This is because the price of a share reflects not only the dividends a company currently pays, but also investors’ expectations of the dividends it will pay in future years. If investors believe the dividends a company pays will go up, they will be willing to pay more to get hold of its shares today, and consequently its share price will rise. If, on the other hand, investors are worried about the future profitability of a company, its share price will fall. Changes in share prices in the present therefore reveal information about the future, making them a useful source of information for understanding how a given event is expected to affect different companies and industries.

In the context of the rapid spread of COVID‐19, changes in share prices reflect market expectations about a number of effects, including changes in final demand (people are buying more of some items and less of others), changes in intermediate demand (the firms that companies sell to are changing what they want to buy and how much) and restrictions in supply (it may be difficult for some firms to obtain inputs they need due, for instance, to interruptions to their supply chain).

Stock market data are available much more rapidly than official data on, for example, unemployment or GDP growth. Indeed, they are updated second by second as investors react to the latest events, making a valuable and timely source of information. They do have a few limitations, however, when it comes to measuring the impact of the crisis. They do not include small firms, firms that are not listed on the stock exchange, the third sector or the public sector, which might be affected quite differently. For example, many public sector services have seen an increase in demand during the crisis. In addition, many firms operate internationally, so changes in their share prices will represent the effects not only on the UK economy but also in other markets that they operate in. Finally, other factors may also have affected share prices over this period. For example, a price war between major oil‐producing countries led to falls in international oil prices and the share prices of fossil fuel companies in late February and March.

Figure 1 shows the percentage change in the price of the FTSE All‐Share price index over the period from 2 January to 20 May 2020. The price index remained pretty steady in the early weeks of the crisis but saw a sharp decline in the weeks following the announcement of a lockdown in northern Italy and fell to its lowest point in the week following the announcement of social distancing in the UK (down 35 per cent from the start of January). As lockdowns have steadily been eased across the world and in the UK, some of this decline has been reversed, taking the overall decline in the FTSE All‐Share Index over the period to 21 per cent.

**FIGURE 1:**
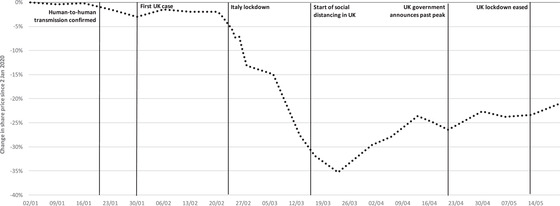
Percentage change in FTSE All‐Share Index, from 2 January to 20 May 2020 *Note*: Authors’ calculations based on indices of sector share prices, as reported on shareprices.com (accessed on 22 May 2020). Vertical lines indicate: 20 January, the first confirmed human‐to‐human transmission in China and first WHO report; 30 January, first confirmed case in the UK; 23 February, Italy introduced lockdown in Lombardy; 16 March, UK Prime Minister first urged social distancing; 21 April, government announced the UK is past peak cases; 13 May, lockdown measures eased.

Figure 2 shows the change in the share price of all firms listed on the London Stock Exchange relative to the FTSE All‐Share Index between 2 January and 20 May 2020. The industries that have been hardest hit include tourism and leisure (which includes air travel), fossil fuels production and distribution, banking, insurance, retailers (excluding food and drug retailers) and some large manufacturing industries. At the other end of the spectrum, some industries have outperformed the market, including food and drug manufacturers and retailers, utilities, high‐tech manufacturing and tobacco. Unsurprisingly, firms in medical and biotech research have also outperformed the market (increasing by 6 per cent compared with the overall decline of 21 per cent).

**FIGURE 2:**
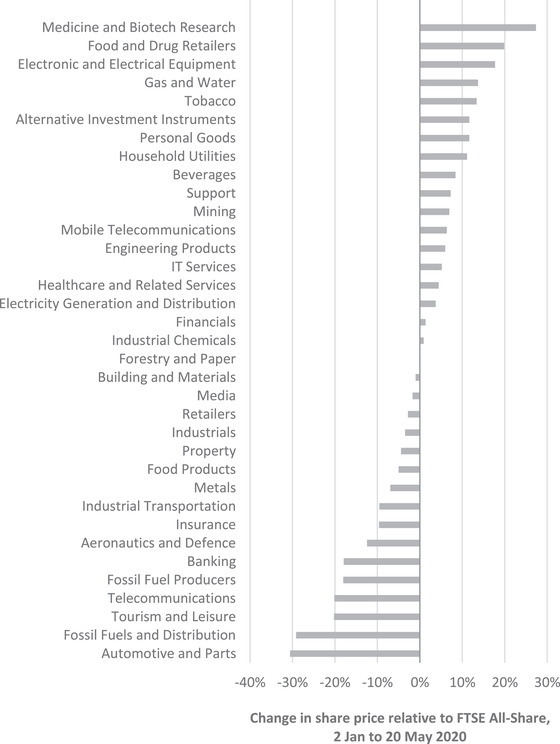
Percentage change in share prices of firms in different sectors listed on the London Stock Exchange relative to the FTSE All‐Share Index, 2 January to 20 May 2020 *Note*: Authors’ calculations based on indices of sector share prices, as reported on shareprices.com (accessed on 22 May 2020), using Industry Classification Benchmark definitions.

The timing of changes in share prices reflects the timing of changes in market expectations. Figure 3 shows the cumulative change in share price over the period for the four sectors with the largest increase and the largest decrease in their relative share price over the period from 2 January to 20 May 2020. For most of these sectors, changes in share prices did not take place steadily over the period. Instead, big changes in share prices occurred from the end of February, in the days following Italy's introduction of a lockdown in Lombardy, with very little change in prices in the period before. The exceptions to this are the gas and water, automotive and parts, and telecommunications industries, where changes in share prices took place steadily over the five‐month period, possibly driven by other factors.

**FIGURE 3:**
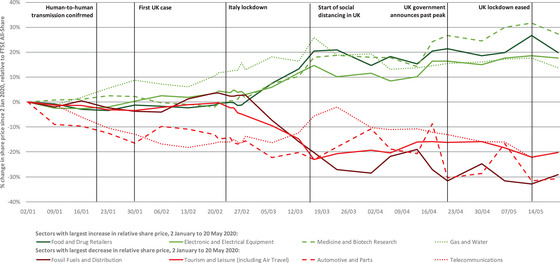
Percentage change in share prices of firms listed on the London Stock Exchange in sectors with the largest share price movements relative to the FTSE All‐Share Index, 2 January to 20 May 2020 *Note*: Authors’ calculations based on indices of sector share prices, as reported on shareprices.com (accessed on 22 May 2020), using Industry Classification Benchmark definitions. Vertical lines indicate: 20 January, the first confirmed human‐to‐human transmission in China and first WHO report; 30 January, first confirmed case in the UK; 23 February, Italy introduced lockdown in Lombardy; 16 March, UK Prime Minister first urged social distancing; 21 April, government announced the UK is past peak cases; 13 May, lockdown measures eased.

On 17 March, the government announced a raft of measures aimed at protecting workers and businesses affected by measures taken to contain the spread of COVID‐19. These included a 12‐month business rates holiday for firms in the retail, leisure and hospitality industries and a Coronavirus Job Retention Scheme (CJRS) that would pay 80 per cent of employee wages for furloughed workers up to a maximum of £2,500 per month for each employee. This package is aimed, at least in part, at preventing otherwise viable businesses from shutting down. The value of these measures will vary greatly across sectors. Labour‐intensive sectors in particular are likely to benefit more from the CJRS than other industries. In other sectors, capital costs and purchases of inputs from other industries are more significant; the benefit to firms in these industries will depend crucially on their ability to adjust their costs as they reduce output. For instance, airlines may use less fuel as more planes are grounded and flights cancelled, but they may still face the costs of leasing and maintaining those planes. The extent to which firms can quickly reduce their costs, and then, when the recovery starts, quickly scale up again, will depend on many factors, such as what these costs are and what sort of supply relationships they have.

To get an idea of the potential benefit of the CJRS for different industries relative to how badly those industries are likely to be affected, Figure 4 plots the potential coverage rates of the CJRS against the cumulative relative share price declines for some of the key industries. The horizontal axis shows the *maximum* extent of support the government has made available via the CJRS, i.e. if every private sector employee working in that industry (not only those in firms listed on the stock market) were furloughed and all firms claimed the full 80 per cent of labour costs up to the £2,500 monthly limit; we scale this by total output of firms in each industry. The support will vary depending on how important labour is as a share of production, compared with machines and intermediate inputs (such as parts and electricity), and depending on the distribution of wages in each industry. The vertical axis shows the size of the relative change in share prices from 2 January to 20 May 2020, as shown in Figure 2. The size of each circle represents the number of people employed in that industry.

**FIGURE 4:**
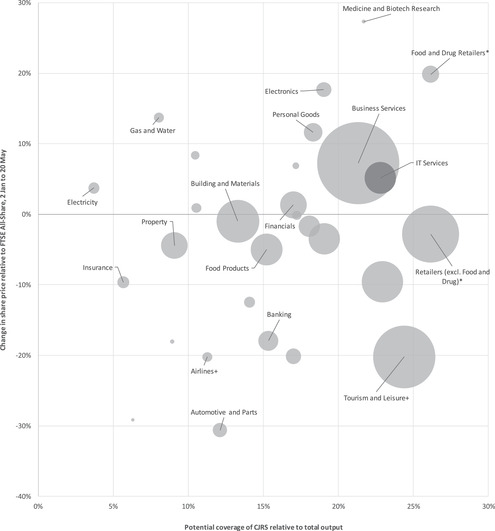
Potential coverage of Coronavirus Job Retention Scheme relative to output for different industries versus their share price changes relative to the FTSE All‐Share Index from 2 January to 20 May 2020 ^*^Potential coverage of the CJRS for food and drug retailers is assumed to be the same as for retailers (excluding food and drug retailers). ^+^We have separated tourism and leisure (excluding air travel) and airlines for the purposes of the horizontal axis, but the share price information is for these two activities combined. *Note*: The vertical axis shows the size of the relative change in share prices from Figure 2. The horizontal axis shows the potential coverage of the CJRS as a share of total industry output. The size of each circle represents the number of people employed in each industry. Potential coverage of the CJRS is 80 per cent of wage costs in each industry up to a maximum of £2,500 per worker per month. To obtain the potential coverage of the scheme relative to output, we multiply the potential coverage of each industry's wage bill by each industry's share of labour costs relative to total output. Labour cost shares are taken from the 2015 ONS input–output tables. The potential coverage of the CJRS is calculated using the 2018 Quarterly Labour Force Survey. Employment data are taken from the 2018 Business Register and Employment Survey. CJRS coverage for medical and biotech research and for banking are taken from industries with Standard Industrial Classification (SIC) codes beginning 64 and 72 respectively (which are also included in the coverage rates for business services and for financials respectively). The graph does not include data for alternative investment instruments, engineering products, household utilities, metals, mobile telecommunications or tobacco, either because these sectors could not be mapped to industries in the input–output tables or because they had negligible employment shares. We exclude healthcare and related services because the majority of employment in this industry is in the public sector.

Figure 4 shows that the support is more effectively targeted at some industries than others. For example, retail, for which labour is a relatively high share of output, has a comparatively high share of output covered by the CJRS. Insurance, airlines, automotive and parts, and building and materials, on the other hand, have relatively small shares covered by the CJRS. Tourism and leisure (excluding air travel) stands out in being one of the hardest‐hit industries (in terms of seeing a large reduction in relative share price) and having a relatively high share of protection through the CJRS.

As social distancing measures continue, more capital‐intensive firms that are not able to substantially reduce their costs may start to struggle more; this may lead to pressure for further government support. There are likely to be long‐run costs to the economy if these firms were forced to shut down and the skills and experience of their workers were lost.
